# Ethyl 2-[(carbamoyl­amino)­imino]­propano­ate hemihydrate

**DOI:** 10.1107/S160053681102530X

**Published:** 2011-07-02

**Authors:** Charlane C. Corrêa, José Eugênio J. C. Graúdo, Luiz Fernando C. de Oliveira, Mauro V. de Almeida, Renata Diniz

**Affiliations:** a Núcleo de Espectroscopia e Estrutura Molecular (NEEM), Department of Chemistry - Federal University of Juiz de Fora - Minas Gerais, 36036-900, Brazil; b Department of Chemistry - Federal University of Juiz de Fora - Minas Gerais, 36036-900, Brazil

## Abstract

The title compound, C_6_H_11_N_3_O_3_·0.5H_2_O, has two independent mol­ecules and one mol­ecule of water in the asymmetric unit. The crystal packing is stabilized by inter­molecular N—H⋯N, O—H⋯O, N—H⋯O and C—H⋯O hydrogen bonds. These inter­actions form a two-dimensional array in the *ab* plane with a zigzag motif which has an angle close to 35° between the zigzag planes. The hydrogen bonding can be best described using the graph-set notation as *N*_1_ = *C*(10)*R*_2_^2^(10)*R*_2_^2^(8) and *N*_2_ = *R*_6_^4^(20)*R*_2_^2^(8).

## Related literature

For the synthesis and applications of ethyl pyruvate semicarbazone, see: Kulka (1946 ▶); Dimmock *et al.* (1993 ▶); Cerecetto *et al.* (2000 ▶); Armor (1992 ▶). For hydrogen-bond motifs, see: Etter *et al.* (1990 ▶). 
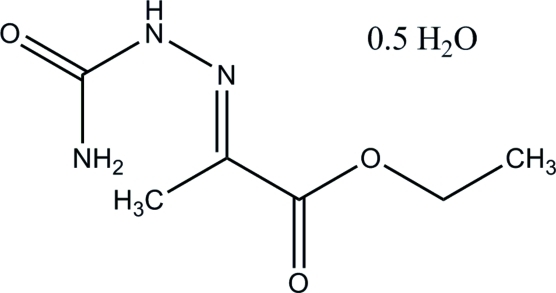


## Experimental

### 

#### Crystal data

C_6_H_11_N_3_O_3_·0.5H_2_O
*M* *_r_* = 182.19
Monoclinic, 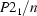

*a* = 11.173 (2) Å
*b* = 14.756 (3) Å
*c* = 11.565 (2) Å
β = 103.14 (3)°
*V* = 1856.8 (6) Å^3^
*Z* = 8
Mo *K*α radiation
μ = 0.11 mm^−1^
*T* = 293 K
0.21 × 0.10 × 0.09 mm

#### Data collection

Nonius KappaCCD diffractometer
18093 measured reflections
4228 independent reflections
1816 reflections with *I* > 2σ(*I*)
*R*_int_ = 0.109

#### Refinement

*R*[*F* ^2^ > 2σ(*F* ^2^)] = 0.059
*wR*(*F* ^2^) = 0.137
*S* = 1.00
4228 reflections
255 parameters
H atoms treated by a mixture of independent and constrained refinement
Δρ_max_ = 0.20 e Å^−3^
Δρ_min_ = −0.18 e Å^−3^

### 

Data collection: *COLLECT* (Hooft, 1999 ▶); cell refinement: *SCALEPACK* (Otwinowski & Minor, 1997 ▶); data reduction: *DENZO* (Otwinowski & Minor 1997 ▶) and *SCALEPACK*; program(s) used to solve structure: *SHELXS97* (Sheldrick, 2008) ▶; program(s) used to refine structure: *SHELXL97* (Sheldrick, 2008) ▶; molecular graphics: *ORTEP-3 for Windows* (Farrugia, 1997 ▶) and *Mercury* (Macrae *et al.*, 2006) ▶; software used to prepare material for publication: *PLATON* (Spek, 2009 ▶).

## Supplementary Material

Crystal structure: contains datablock(s) global, I. DOI: 10.1107/S160053681102530X/qm2011sup1.cif

Structure factors: contains datablock(s) I. DOI: 10.1107/S160053681102530X/qm2011Isup2.hkl

Supplementary material file. DOI: 10.1107/S160053681102530X/qm2011Isup3.cml

Additional supplementary materials:  crystallographic information; 3D view; checkCIF report

## Figures and Tables

**Table 1:** Hydrogen-bond geometry (Å, °)

*D*—H⋯*A*	*D*—H	H⋯*A*	*D*⋯*A*	*D*—H⋯*A*
N4—H4*N*1⋯O1	0.85 (3)	2.19 (3)	3.030 (3)	166 (3)
N1—H1*N*1⋯O4	0.86 (3)	2.14 (3)	2.989 (3)	169 (3)
N2—H2*N*⋯O4^i^	0.84 (2)	2.09 (2)	2.920 (3)	169 (2)
N4—H4*N*2⋯O7^ii^	0.92 (3)	2.05 (3)	2.950 (3)	169 (3)
N1—H1*N*2⋯N3	0.88 (3)	2.31 (3)	2.656 (3)	103.5 (18)
N1—H1*N*2⋯O7	0.88 (3)	2.10 (3)	2.955 (3)	163 (3)
N5—H5*N*⋯O1^iii^	0.84 (2)	2.11 (2)	2.935 (3)	167 (2)
O7—H7*A*⋯O2	0.92	1.92	2.809 (3)	163
O7—H7*B*⋯O5^iv^	0.88	2.01	2.826 (3)	153
C6—H6*C*⋯O4^i^	0.96	2.47	3.397 (3)	162

Symmetry codes: (i) 
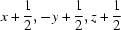
; (ii) 
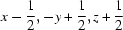
; (iii) 
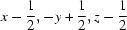
; (iv) 
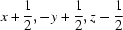
.

**Fig. 1.**
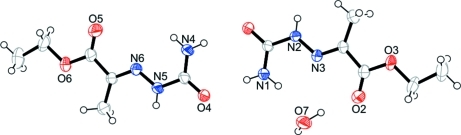
View ORTEP of crystal structure of compound spe.

**Fig. 2.**
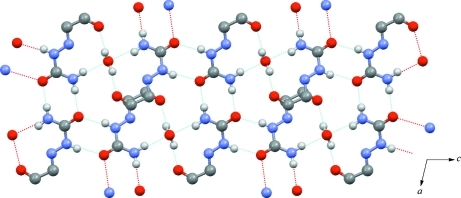
Hydrogen bonds between molecules of ethyl pyruvate semicarbazone and the water molecule, elucidating the ring formed by hydrogen bonds.

**Fig. 3.**
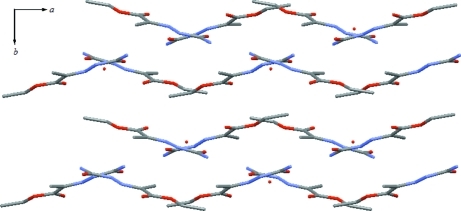
Crystal packing of compound spe, viewed along the c axis.

## supplementary crystallographic information

### Comment

Semicarbazones have been important in the consistent advances in design of novel anticonvulsant agents through the work of Dimmock *et al.* (Dimmock,1993); the aryl semicarbazone 4-bromobenzaldehyde semicarbazone has been a standard anticonvulsant drug selected by the National Institutes of Health, USA. They can also be used as drugs for Chagas's disease (Cerecetto, 2000) as well as hypnotic, pesticide and herbicide uses (Armor, 1992). The synthesis of ethyl pyruvate semicarbazone (spe) (Scheme I) was reported more than 60 years (Kulka, 1946); but to date the structure determination has not been reported. This structure will permit further study of the metal coordination properties of these interesting ligands.

Figure 1 shows the *ORTEP* representation of the asymmetric unit of spe. There are two crystallographically independent molecules and one water of crystallization in the asymmetric unit. The main differences between these molecules are the distances involved in the hydrogen bonds between [O4···H1N1···N1 = 2.989 (3) Å; O1···H4N1···N4 = 3.030 (3) Å; O7···H1N2···N1 = 2.955 (3) Å and O7^i^···H4N2···N4 = 2.950 (3) Å] *(symmetry code: i: x - 1/2, -y + 1/2, z + 1/2)* and also by the torsion angles between the groups CH_3_ and CH_2_ [C10—O6—C9—O5 = 0.8 (4)° and C4—O3—C3—O2 =-1.3 (4)°; C10—O6—C9—C8 = -179.00 (2)° and C4—O3—C3—C2 = 179,60 (2)°; C12—C8—C9—O5 = -178.90 (3)° and C6—C2—C3—O2 = - 173.10 (3)°; C12—C8—C9—O6 = 1.0 (4)° and C6—C2—C3—O3 = 6.0 (3)°]. The water molecule of crystallization stabilizes the crystal lattice by the intermolecular hydrogen bonds N—H···N, O—H···O and O—H···N, with average distances between the electronegative atoms of: 2.656 (3), 2.818 (3), 2.963 (3) Å, respectively. The hydrogen bonding scheme is displayed in Figure 2 and using the graph-set notation the system can be best described as N_1_=C(10)*R*_2_^2^(10)*R*_2_^2^(8) and N_2_=*R*_6_^4^(20)*R*_2_^2^(8) (Etter, 1990).

There are two isomeric forms for compounds derived from ethyl pyruvate semicarbazone namely the *E* and *Z* isomers. The present structure is clearly the *E* form, Figures 1 and 2. The molecules of spe form a two-dimensional array in the *ab* plane with a zigzag motif which has an angle close to 35° between the zigzag planes.

### Experimental

For the preparation of ethyl pyruvate semicarbazone spe, 50 ml of an aqueous solution of semicarbazide chloride (1.019 g, 9.14 mmol) was added to a 50 ml of an ethanolic solution of ethylpyruvate (1.060 g, 9.14 mmol) and amonium acetate (0.744 g, 9.66 mmol), and the final mixture was heated at 80 °C for 6 h. Colorless crystals were formed. The compound decomposes at 175 °C. Elemental analysis gave the following results: Anal. Calcd. for C_6_H_13_O_4_N_3_: C,37,69; H,6,85; N,21,98%; Found: C,38,33; H,5,85; N,22,09%. Infrared spectra show absorption bands at 3506–3170 cm^-1^ (νNH + νOH); 1698 cm^-1^ (νCO); 1599 cm^-1^ (νNH + νCN + νCC); 1480 cm^-1^(νCOasym); 1357 cm^-1^ (νCN); 1144 cm^-1^ (νCOsym) and 1103 cm^-1^ (νCOC).

### Refinement

C-bound H atoms were included using the riding model approximation with C—H = 0.95 Å, with a common *U*_iso_(H). The H atoms of the water molecule were located from an electron density map, fixed in these positions and assigned the same isotropic displacement as the other H atoms.

### Crystal data

**Table:**

C_6_H_11_N_3_O_3_·0.5H_2_O	*F*(000) = 776
*M**_r_* = 182.19	*D*_x_ = 1.303 Mg m^−^^3^
Monoclinic, *P*2_1_/*n*	Mo *K*α radiation, λ = 0.71073 Å
Hall symbol: -P 2yn	Cell parameters from 33 reflections
*a* = 11.173 (2) Å	θ = 4.5–18.2°
*b* = 14.756 (3) Å	µ = 0.11 mm^−^^1^
*c* = 11.565 (2) Å	*T* = 293 K
β = 103.14 (3)°	Prism, colourless
*V* = 1856.8 (6) Å^3^	0.21 × 0.10 × 0.09 mm
*Z* = 8	

### Data collection

**Table:**

Nonius KappaCCD diffractometer	1816 reflections with *I* > 2σ(*I*)
Radiation source: fine-focus sealed tube	*R*_int_ = 0.109
horizonally mounted graphite crystal	θ_max_ = 27.5°, θ_min_ = 5.2°
Detector resolution: 9 pixels mm^-1^	*h* = −13→14
CCD scans	*k* = −18→19
18093 measured reflections	*l* = −15→14
4228 independent reflections	

### Refinement

**Table:**

Refinement on *F*^2^	Hydrogen site location: inferred from neighbouring sites
Least-squares matrix: full	H atoms treated by a mixture of independent and constrained refinement
*R*[*F*^2^ > 2σ(*F*^2^)] = 0.059	*w* = 1/[σ^2^(*F*_o_^2^) + (0.045*P*)^2^ + 0.5582*P*] where *P* = (*F*_o_^2^ + 2*F*_c_^2^)/3
*wR*(*F*^2^) = 0.137	(Δ/σ)_max_ < 0.001
*S* = 1.00	Δρ_max_ = 0.20 e Å^−^^3^
4228 reflections	Δρ_min_ = −0.18 e Å^−^^3^
255 parameters	Extinction correction: *SHELXL97* (Sheldrick, 2008), Fc^*^=kFc[1+0.001xFc^2^λ^3^/sin(2θ)]^-1/4^
0 restraints	Extinction coefficient: 0.0059 (11)
Primary atom site location: structure-invariant direct methods	Absolute structure: no
Secondary atom site location: difference Fourier map	

### Special details

**Table:**

Geometry. All e.s.d.'s (except the e.s.d. in the dihedral angle between two l.s. planes) are estimated using the full covariance matrix. The cell e.s.d.'s are taken into account individually in the estimation of e.s.d.'s in distances, angles and torsion angles; correlations between e.s.d.'s in cell parameters are only used when they are defined by crystal symmetry. An approximate (isotropic) treatment of cell e.s.d.'s is used for estimating e.s.d.'s involving l.s. planes.
Refinement. Refinement of *F*^2^ against ALL reflections. The weighted *R*-factor *wR* and goodness of fit *S* are based on *F*^2^, conventional *R*-factors *R* are based on *F*, with *F* set to zero for negative *F*^2^. The threshold expression of *F*^2^ > σ(*F*^2^) is used only for calculating *R*-factors(gt) *etc*. and is not relevant to the choice of reflections for refinement. *R*-factors based on *F*^2^ are statistically about twice as large as those based on *F*, and *R*- factors based on ALL data will be even larger.

### Fractional atomic coordinates and isotropic or equivalent isotropic displacement parameters (Å^2^)

**Table:**

	*x*	*y*	*z*	*U*_iso_*/*U*_eq_	
N6	0.63805 (17)	0.15088 (15)	0.54625 (17)	0.0361 (5)	
O4	0.88839 (14)	0.22111 (13)	0.43482 (14)	0.0462 (5)	
O1	1.12914 (15)	0.27042 (14)	0.71551 (14)	0.0482 (5)	
N5	0.70387 (18)	0.17912 (16)	0.4671 (2)	0.0387 (6)	
N2	1.31214 (18)	0.31483 (16)	0.6804 (2)	0.0394 (6)	
N3	1.37693 (17)	0.34035 (15)	0.59917 (16)	0.0347 (5)	
O6	0.34452 (15)	0.08529 (13)	0.56959 (15)	0.0493 (5)	
O3	1.66804 (14)	0.40150 (13)	0.56711 (15)	0.0451 (5)	
O7	1.25278 (16)	0.33845 (15)	0.32243 (15)	0.0577 (6)	
H7A	1.3224	0.3672	0.3643	0.087*	
H7B	1.1933	0.3754	0.2876	0.087*	
C8	0.5218 (2)	0.13447 (18)	0.5091 (2)	0.0339 (6)	
N1	1.1427 (2)	0.29295 (18)	0.52453 (19)	0.0458 (7)	
C1	1.1895 (2)	0.29159 (18)	0.6407 (2)	0.0337 (6)	
C2	1.4919 (2)	0.36056 (17)	0.6338 (2)	0.0333 (6)	
C7	0.8274 (2)	0.19917 (18)	0.5083 (2)	0.0370 (7)	
O5	0.51752 (17)	0.09562 (17)	0.71042 (17)	0.0733 (7)	
O2	1.49015 (16)	0.39939 (15)	0.42999 (16)	0.0612 (6)	
N4	0.8733 (2)	0.19408 (19)	0.6242 (2)	0.0503 (7)	
C9	0.4642 (2)	0.10351 (19)	0.6078 (2)	0.0420 (7)	
C3	1.5468 (2)	0.38847 (19)	0.5318 (2)	0.0397 (7)	
C10	0.2801 (2)	0.0561 (2)	0.6597 (3)	0.0576 (9)	
H10A	0.2850	0.1026	0.7199	0.069*	
H10B	0.3167	0.0010	0.6978	0.069*	
C12	0.4459 (2)	0.1434 (2)	0.3850 (2)	0.0542 (8)	
H12A	0.4931	0.1239	0.3298	0.081*	
H12B	0.4223	0.2056	0.3696	0.081*	
H12C	0.3736	0.1065	0.3759	0.081*	
C11	0.1485 (3)	0.0396 (2)	0.5977 (3)	0.0725 (10)	
H11A	0.1141	0.0941	0.5584	0.109*	
H11B	0.1026	0.0221	0.6549	0.109*	
H11C	0.1446	−0.0079	0.5403	0.109*	
C5	1.8633 (3)	0.4440 (2)	0.5311 (3)	0.0700 (10)	
H5A	1.8705	0.4941	0.5852	0.105*	
H5B	1.9075	0.4575	0.4709	0.105*	
H5C	1.8971	0.3906	0.5735	0.105*	
C4	1.7302 (2)	0.4283 (2)	0.4739 (3)	0.0533 (8)	
H4A	1.6936	0.4833	0.4354	0.064*	
H4B	1.7223	0.3809	0.4145	0.064*	
C6	1.5688 (2)	0.3594 (2)	0.7578 (2)	0.0595 (9)	
H6A	1.6120	0.4159	0.7742	0.089*	
H6B	1.6270	0.3106	0.7661	0.089*	
H6C	1.5169	0.3509	0.8126	0.089*	
H2N	1.344 (2)	0.3065 (15)	0.753 (2)	0.026 (7)*	
H4N1	0.949 (3)	0.2061 (19)	0.653 (2)	0.061 (9)*	
H1N1	1.066 (3)	0.2794 (19)	0.501 (2)	0.055 (9)*	
H5N	0.672 (2)	0.1881 (19)	0.395 (2)	0.056 (9)*	
H4N2	0.826 (3)	0.1839 (18)	0.678 (2)	0.051 (8)*	
H1N2	1.190 (3)	0.3057 (18)	0.475 (2)	0.048 (8)*	

### Atomic displacement parameters (Å^2^)

**Table:**

	*U*^11^	*U*^22^	*U*^33^	*U*^12^	*U*^13^	*U*^23^
N6	0.0261 (11)	0.0499 (15)	0.0330 (12)	−0.0012 (10)	0.0084 (9)	0.0002 (10)
O4	0.0291 (9)	0.0795 (15)	0.0315 (10)	−0.0106 (10)	0.0100 (8)	−0.0059 (10)
O1	0.0293 (9)	0.0856 (16)	0.0318 (10)	−0.0126 (10)	0.0115 (8)	−0.0038 (10)
N5	0.0262 (11)	0.0616 (17)	0.0279 (12)	−0.0037 (11)	0.0054 (10)	−0.0026 (12)
N2	0.0243 (11)	0.0687 (18)	0.0248 (12)	−0.0053 (11)	0.0048 (9)	0.0055 (12)
N3	0.0262 (11)	0.0472 (15)	0.0316 (12)	−0.0042 (10)	0.0082 (9)	0.0021 (10)
O6	0.0295 (9)	0.0687 (14)	0.0507 (11)	−0.0117 (10)	0.0113 (8)	0.0018 (10)
O3	0.0249 (9)	0.0636 (14)	0.0499 (11)	−0.0033 (9)	0.0150 (8)	0.0059 (10)
O7	0.0378 (10)	0.0966 (16)	0.0365 (9)	0.0016 (11)	0.0036 (8)	−0.0013 (11)
C8	0.0269 (13)	0.0424 (17)	0.0326 (14)	0.0004 (12)	0.0071 (11)	0.0002 (12)
N1	0.0259 (12)	0.081 (2)	0.0305 (13)	−0.0111 (13)	0.0063 (11)	−0.0008 (12)
C1	0.0244 (13)	0.0485 (18)	0.0287 (14)	−0.0020 (12)	0.0071 (11)	−0.0032 (12)
C2	0.0227 (12)	0.0410 (17)	0.0366 (14)	0.0009 (12)	0.0073 (10)	0.0007 (12)
C7	0.0266 (13)	0.0505 (19)	0.0336 (15)	−0.0015 (13)	0.0062 (12)	−0.0056 (13)
O5	0.0409 (11)	0.132 (2)	0.0447 (12)	−0.0193 (13)	0.0040 (10)	0.0239 (13)
O2	0.0401 (11)	0.1044 (18)	0.0385 (11)	−0.0128 (12)	0.0076 (9)	0.0136 (11)
N4	0.0290 (13)	0.093 (2)	0.0281 (13)	−0.0114 (13)	0.0049 (11)	−0.0015 (13)
C9	0.0288 (14)	0.052 (2)	0.0444 (17)	−0.0046 (13)	0.0061 (12)	0.0043 (14)
C3	0.0295 (14)	0.0485 (19)	0.0423 (16)	−0.0007 (13)	0.0107 (12)	0.0012 (14)
C10	0.0437 (17)	0.071 (2)	0.065 (2)	−0.0103 (16)	0.0258 (15)	0.0107 (17)
C12	0.0320 (15)	0.084 (2)	0.0445 (17)	−0.0064 (15)	0.0040 (13)	0.0101 (16)
C11	0.0404 (17)	0.077 (3)	0.106 (3)	−0.0113 (17)	0.0272 (18)	0.005 (2)
C5	0.0455 (17)	0.081 (3)	0.093 (2)	−0.0205 (17)	0.0336 (17)	−0.013 (2)
C4	0.0407 (16)	0.061 (2)	0.066 (2)	−0.0024 (15)	0.0293 (15)	0.0131 (16)
C6	0.0288 (14)	0.104 (3)	0.0435 (17)	−0.0072 (16)	0.0042 (13)	0.0098 (17)

### Geometric parameters (Å, °)

**Table:**

N6—C8	1.295 (3)	O7—H7B	0.8700
N6—N5	1.363 (3)	N1—H1N1	0.86 (3)
O4—C7	1.246 (3)	N1—H1N2	0.88 (3)
O1—C1	1.252 (3)	N2—H2N	0.84 (2)
N5—C7	1.386 (3)	N5—H5N	0.84 (2)
N2—N3	1.363 (3)	N4—H4N1	0.85 (3)
N2—C1	1.386 (3)	N4—H4N2	0.92 (3)
N3—C2	1.291 (3)	C5—H5C	0.9600
O6—C9	1.337 (3)	C5—H5A	0.9600
O6—C10	1.460 (3)	C5—H5B	0.9600
O3—C3	1.337 (3)	C4—H4A	0.9700
O3—C4	1.463 (3)	C4—H4B	0.9700
C8—C12	1.498 (3)	C6—H6C	0.9600
C8—C9	1.503 (3)	C6—H6A	0.9600
N1—C1	1.326 (3)	C6—H6B	0.9600
C2—C6	1.495 (3)	C11—H11A	0.9600
C2—C3	1.505 (3)	C11—H11B	0.9600
C7—N4	1.324 (3)	C11—H11C	0.9600
O5—C9	1.207 (3)	C10—H10A	0.9700
O2—C3	1.215 (3)	C10—H10B	0.9700
C10—C11	1.502 (4)	C12—H12A	0.9600
C5—C4	1.502 (4)	C12—H12B	0.9600
O7—H7A	0.9100	C12—H12C	0.9600
			
O1···N5^i^	2.935 (3)	O5···O7^iv^	2.826 (3)
O1···C12^i^	3.388 (3)	O7···O5^v^	2.826 (3)
O1···N4	3.030 (3)	O7···N6^v^	3.164 (3)
O2···C3^ii^	3.201 (4)	O7···O2	2.809 (3)
O2···O7	2.809 (3)	O7···N1	2.955 (3)
O2···N3	2.704 (3)	O7···N3	3.186 (3)
O4···N1	2.989 (3)	O7···N4^v^	2.950 (3)
O4···C6^iii^	3.397 (3)	N1···N3	2.656 (3)
O4···N2^iii^	2.920 (3)	N4···N6	2.655 (3)
O5···N6	2.692 (3)		
			
C8—N6—N5	119.2 (2)	C7—N4—H4N1	120.3 (16)
N6—N5—C7	118.8 (2)	C7—N4—H4N2	123.1 (16)
N3—N2—C1	118.7 (2)	H5A—C5—H5B	109.00
C2—N3—N2	119.8 (2)	H5B—C5—H5C	109.00
C9—O6—C10	116.3 (2)	H5A—C5—H5C	110.00
C3—O3—C4	115.6 (2)	C4—C5—H5C	109.00
N6—C8—C12	127.4 (2)	C4—C5—H5B	109.00
N6—C8—C9	112.1 (2)	O3—C4—H4A	110.00
C12—C8—C9	120.5 (2)	O3—C4—H4B	110.00
O1—C1—N1	123.6 (2)	H4A—C4—H4B	109.00
O1—C1—N2	118.7 (2)	C5—C4—H4A	110.00
N1—C1—N2	117.7 (2)	C5—C4—H4B	110.00
N3—C2—C6	127.6 (2)	C2—C6—H6C	109.00
N3—C2—C3	112.0 (2)	H6B—C6—H6A	109.00
C6—C2—C3	120.4 (2)	C2—C6—H6B	109.00
O4—C7—N4	124.0 (2)	H6B—C6—H6C	110.00
O4—C7—N5	118.5 (2)	H6A—C6—H6C	109.00
N4—C7—N5	117.5 (2)	C2—C6—H6A	109.00
O5—C9—O6	122.6 (2)	C10—C11—H11A	109.00
O5—C9—C8	125.1 (2)	C10—C11—H11B	109.00
O6—C9—C8	112.3 (2)	H11A—C11—H11B	109.00
O2—C3—O3	123.1 (2)	H11A—C11—H11C	109.00
O2—C3—C2	125.5 (2)	H11B—C11—H11C	109.00
O3—C3—C2	111.4 (2)	C10—C11—H11C	110.00
O6—C10—C11	107.2 (2)	O6—C10—H10A	110.00
O3—C4—C5	107.8 (2)	C11—C10—H10A	110.00
H7A—O7—H7B	114.00	C11—C10—H10B	110.00
C1—N1—H1N2	120.3 (18)	O6—C10—H10B	110.00
H1N1—N1—H1N2	123 (2)	C8—C12—H12B	109.00
C1—N1—H1N1	116.8 (16)	C8—C12—H12C	109.00
C1—N2—H2N	117.1 (16)	C8—C12—H12A	109.00
N3—N2—H2N	123.8 (16)	H12A—C12—H12C	109.00
C7—N5—H5N	118.2 (16)	H12B—C12—H12C	109.00
N6—N5—H5N	122.9 (16)	H12A—C12—H12B	110.00
			
C4—O3—C3—O2	−1.3 (4)	N6—N5—C7—O4	176.9 (2)
C4—O3—C3—C2	179.6 (2)	N6—N5—C7—N4	−3.4 (4)
C3—O3—C4—C5	177.3 (2)	N5—N6—C8—C9	179.6 (2)
C10—O6—C9—C8	−179.0 (2)	N5—N6—C8—C12	−0.5 (4)
C9—O6—C10—C11	180.0 (3)	N3—C2—C3—O2	6.4 (4)
C10—O6—C9—O5	0.8 (4)	C6—C2—C3—O2	−173.1 (3)
C1—N2—N3—C2	−178.6 (2)	C6—C2—C3—O3	6.0 (3)
N3—N2—C1—O1	−178.2 (2)	N3—C2—C3—O3	−174.5 (2)
N3—N2—C1—N1	2.0 (4)	N6—C8—C9—O5	1.0 (4)
N2—N3—C2—C6	0.4 (4)	N6—C8—C9—O6	−179.1 (2)
N2—N3—C2—C3	−179.1 (2)	C12—C8—C9—O5	−178.9 (3)
C7—N5—N6—C8	178.1 (2)	C12—C8—C9—O6	1.0 (4)

Symmetry codes: (i) *x*+1/2, −*y*+1/2, *z*+1/2; (ii) −*x*+3, −*y*+1, −*z*+1; (iii) *x*−1/2, −*y*+1/2, *z*−1/2; (iv) *x*−1/2, −*y*+1/2, *z*+1/2; (v) *x*+1/2, −*y*+1/2, *z*−1/2.

### Hydrogen-bond geometry (Å, °)

**Table:**

*D*—H···*A*	*D*—H	H···*A*	*D*···*A*	*D*—H···*A*
N4—H4N1···O1	0.85 (3)	2.19 (3)	3.030 (3)	166 (3)
N1—H1N1···O4	0.86 (3)	2.14 (3)	2.989 (3)	169 (3)
N2—H2N···O4^i^	0.84 (2)	2.09 (2)	2.920 (3)	169 (2)
N4—H4N2···O7^iv^	0.92 (3)	2.05 (3)	2.950 (3)	169 (3)
N1—H1N2···N3	0.88 (3)	2.31 (3)	2.656 (3)	103.5 (18)
N1—H1N2···O7	0.88 (3)	2.10 (3)	2.955 (3)	163 (3)
N5—H5N···O1^iii^	0.84 (2)	2.11 (2)	2.935 (3)	167 (2)
O7—H7A···O2	0.92	1.92	2.809 (3)	163
O7—H7B···O5^v^	0.88	2.01	2.826 (3)	153
C6—H6C···O4^i^	0.96	2.47	3.397 (3)	162
C6—H6C···N2	0.96	2.50	2.879 (3)	103
C12—H12C···O6	0.96	2.36	2.770 (3)	105

Symmetry codes: (i) *x*+1/2, −*y*+1/2, *z*+1/2; (iv) *x*−1/2, −*y*+1/2, *z*+1/2; (iii) *x*−1/2, −*y*+1/2, *z*−1/2; (v) *x*+1/2, −*y*+1/2, *z*−1/2.
